# Mental health first aid training among healthcare French students: a qualitative study

**DOI:** 10.3389/fmed.2023.1268277

**Published:** 2023-10-23

**Authors:** Jordan Sibeoni, Pierre Ellul, Théo Bubola, Yanis Debiche, Marie-Aude Piot

**Affiliations:** ^1^Argenteuil Hospital Centre, Service Universitaire de Psychiatrie de l’Adolescent, Argenteuil, France; ^2^ECSTRRA Team, UMR, Inserm, Université Paris Cité, Paris, France; ^3^Child and Adolescent Psychiatry Department, Robert Debré Hospital, APHP, Université Paris Cité, Paris, France; ^4^Inserm Immunology-Immunopathology-Immunotherapy (i3), UMRS, Sorbonne Université, Paris, France; ^5^Health Faculty, Medical School, Université Paris Cité, Paris, France; ^6^Department of Child and Adolescent Psychiatry, Hôpital Necker-Enfants–Malade, AP–HP, Paris, France; ^7^Inserm, Centre d’épidémiologie et de santé des Populations (CESP), UMR, USQV, Villejuif, France

**Keywords:** mental health first aid, students, medical education, qualitative research, literacy

## Abstract

**Background:**

Healthcare students are a population more at risk for mental health issues, especially anxiety, depression, and suicidal thoughts. The health faculty of Université Paris Cité in France, Paris has implemented a Mental Health First Aid (MHFA) course aiming to improve students’ mental health literacy, self-care and peer-support and to decrease stigma about mental illness. We conducted a qualitative study exploring the lived experience of this MHFA training course among healthcare students so to better assess its implementation within this specific context and population.

**Methods:**

This qualitative study used the five-stage inductive process to analyze the structure of lived experience (IPSE) approach. All the healthcare students that had completed the 2-day MHFA training were approached to participate. Data was collected through individual semi-structured interviews and inclusion continued until data saturation was reached. Data analysis was based on an inductive, descriptive, and structuring procedure to determine the structure of lived experience characterized by the central axes of experience.

**Results:**

Twenty students were included. Data analysis produced a common structure of lived experience based on three central axes of experience, (1) a personal experience, (2) a student experience and (3) a professional experience. The participants all experienced this course intertwined within these 3 axes. Their motivation to take the course was personal -being of feeling concerned by the topic-, was study-oriented – to learn and revise psychiatry- and was professional – so to develop both practical and soft skills. In their personal experience, participants reported a transformative experience and some interventions with friends and family, while both in their student and professional experience, they felt frustrated with both the content and the form of the course.

**Conclusion:**

The results reported similar outcomes reported in the literature about skills, knowledge, and awareness; but mostly produce original avenues about how to better adapt such course to this specific population so to better address students’ expectations and mental health issues. This MHFA course -with an adapted content addressing eating disorders, self-mutilations and sexual and gender-based violence – could be part of the early curriculum of healthcare students. The latter could then benefit from a level 2/advanced MHFA course years later specifically tailored for healthcare professionals.

## Introduction

The COVID 19’s pandemic shed light on an ongoing major public health issue: the mental health of students in general (1) and particularly medical and healthcare students with specific stressors- relational, financial, accommodations issues- identified (2). A meta-analysis found high prevalence of psychiatric complication among medical students with −52% of sleep disorder, 41% of depression, 38% of anxiety, 38% of burnout and 15% of suicidal ideation (3). A French survey (4) conducted between May and June 2021 among 11,754 French medical students confirmed high levels of prevalence of psychiatric symptoms with one fourth of the respondents presenting a major depressive episode, and one fifth suicidal thoughts in the last 12 months. In our university- Université Paris Cité-, a similar survey was performed but encompassing not only medical but also dental and pharmacy students (*N* = 1925), with similar prevalence of anxiety symptoms (55%), 7-day depressive symptoms (23%), 12-month prevalence of MDE (26%) and 12-month prevalence suicidal thoughts (19%)- (5). Moreover, many students are reluctant to seek professional help, with a poorer utilization of mental health services (6). This reluctance is associated with stigma and a “culture of shame” within this specific population (7). A metasynthesis reported some stigmatizing views from students judging the behavior of individuals with mental illnesses as “*deviant, disorderly, incompetent, or nonconforming*” (8). The issue of training in mental healthcare was frequently addressed, participants feeling unprepared to provide effective care, operating “*beyond their scope of training*.” insufficient and inadequate education contribute to low confidence to provide effective care and to maintain negative perceptions about mental illness (8). On the contrary, interventions increasing mental health literacy among students appeared to be an effective way to promote mental health and to improve recognition and help-seeking efficacy (9). Similarly, another study on US college students found a positive association between mental health literacy and self-compassion (10).

To address globally this public health concern, the health faculty of UPC has implemented an innovative education response by delivering Mental Health First Aid (MHFA) training to medical and healthcare students since April 2022. The French MHFA training program is an adaptation of the original Australian program -translated and tailored to fit with French culture and health system (11) – based on evidence-based medicine and expert consensus guidelines through Delphi method. MHFA training teaches participants how to offer help “to a person developing a mental health problem, experiencing a worsening of an existing mental health problem or in a mental health crisis” (WPA). Many quantitative studies have shown the effectiveness of MHFA training: to improve mental health literacy and appropriate support in the general population (12); to increase knowledge and confidence to support individuals with mental health issues among college students (13), to increase mental health literacy and empower paramedic students to act (14); to improve attitudes, knowledge and decrease stigmatizing beliefs among pharmacists and pharmacy students (15, 16). Several qualitative studies have explored the lived experience and perception of the course participants. For the general population, these studies came from Australia (17, 18), United Kingdom (19) and Sweden (20) exploring mostly through semi-structured interviews the impact and outcomes of the two-day MHFA training. Their results highlighted the increased confidence, helping experiences and empathy of the participants as well as a sense of responsibility and more concerns about their own well-being. A qualitative study from China (21) among previous participants focused on the implementation of MHFA in this country, addressing contextual issues such as adaptation of the content and financing models. Another study from Wales explored the experiences of instructors delivering the training (22) and underlined the emotional labor experienced by them. Finally, two studies focused on the lived experience of students. Rodgers et al. (23) conducted a mixed-method study among Australian university students, with a qualitative phase of 9 interviews highlighting the improved awareness of mental health issues by the students but also how helping someone with such issues was a challenging experience.

To our knowledge, the only qualitative study focusing on healthcare students was conducted in Hong Kong among 25 nursing students, exploring their views about the MHFA training (24). These students described an improvement of their knowledge, skills, and self-awareness as well as some corrections in terms of false beliefs and prejudices (24). No qualitative study has yet explored this topic, neither among healthcare students more globally, nor in France.

Qualitative research is a relevant approach in implementation research to explore an intervention – or here a training course- focusing on the process and effectiveness but also on the context in which and the environment where it occurs (25). This qualitative inductive study aimed to explore the lived experience of French healthcare students of a MHFA 2-day training course recently implemented in the health faculty of UPC, in order to gain some original insights about implementation outcomes – such as feasibility, acceptability, adoption, appropriateness, penetration and sustainability- of this program and the potential adaptations to make among this specific population to reach such outcomes.

## Methods

This exploratory qualitative study used the Inductive Process to analyze the Structure of lived Experience (IPSE) approach (26). IPSE is a 5 stages-approach informed by a descriptive phenomenological approach and relies on an inductive process that explores in depth the lived experience of participants and analyzes the structure of their experiences. The report of this study adheres to the COREQ guidelines (27). The research complies with French regulations governing observational research involving students (declaration of compliance with the CNIL reference methodology MR004 and entry in the register of such research hosted by Health Data Hub website). All procedures involving participants were performed with relevant guidelines and regulations, that is in accordance with the declaration of Helsinki regarding the principles for medical research involving human subjects. All the participants provided written informed consent.

### The 2-day MHFA course

The MHFA course has four pedagogical objectives: to acquire basic knowledge of the most common mental health disorders (depressive disorders, anxiety disorders, psychotic disorders, and substance use disorders); to gain a better understanding of the different types of mental health crises; to develop interpersonal skills; and to better cope with aggressive behavior. It lasts 2 days, with 16 participants divided over the 2 days into groups of four, to encourage discussion. The course content is detailed in Table 1. The course is based on a participative pedagogical approach, alternating theoretical lectures, fictional videos and real-life testimonials, with practical exercises in small groups (role-playing scenarios, group tasks) to apply a method for intervening when faced with a person in crisis or difficulty, the MHFA plan of action that is to (i) approach, assess and assist in case of crisis; (ii) listen and communicate nonjudgmentally; (iii) give support and information; (iv) encourage appropriate professional help; and (v) encourage other supports (28).

**Table 1:** MHFA 2-day course content.

Introduction to mental health	Definition and current situation: risk factors, prevalence, consequences Effective interventions
Introduction to MHFA	The framework and context of MHFA The PSSM action plan
Depression	Signs and symptoms Interventions MHFA action plans First aid for suicidal thoughts and intentions First aid for depression
Anxiety disorders	Signs and symptoms Interventions MHFA action plans First aid for panic attacks First aid after a traumatic event First aid for anxiety disorders
Psychotic disorders	Signs and symptoms Interventions MHFA action plans First aid for severe psychotic episodes First aid for psychotic disorders
Substance use disorders	Signs and symptoms Interventions MHFA action plans First aid for substance poisoning. First aid for aggressive behavior First aid for medical emergencies First Aid for Substance Use Disorder

The health faculty advertised about this training opportunity- optional and free of charge- in a monthly newsletter distributed to all the students through e-mails. Interested students then registered for the different sessions through the year. At the time when the study recruitment took place, 153 students volunteered and undertook the 2-day training course.

### Stage 1: setting up research group

Our research group included three psychiatrists and two medical students, all instructors in MHFA training. The train-the-trainer course takes 5 days, and participants are required to have completed the Mental Health First Aid course, as well as having theoretical, practical and/or experiential knowledge of mental disorders, such as being a mental health professional or expert patient. The course is based on theoretical presentations and practical exercises, enabling participants to “experience MHFAA training as an informed participant,” “explore the tools and practices available for conducting training” and “practice conducting Standard Mental Health First Aid training” (29).

For heuristic purposes—that is, to enable to discover new unknown elements and to produce original findings—the group’s members were highly diverse, especially in their knowledge, age, gender, and backgrounds. The group worked continuously on reflexivity during open discussions among themselves.

### Stage 2: ensuring the originality of the study

Two members of the group reviewed the qualitative and quantitative literature systematically, to confirm the study’s relevance and originality. They verified that no qualitative study of this specific topic had been conducted within a similar context. To ensure that the other group members could remain inductive and open to novelty, they had access to this review only after they had completed the data analysis.

### Stage 3: recruitment and sampling

The research group defined the inclusion and exclusion criteria (Table 2). To facilitate the recruitment process and produce findings rapidly so to make relevant changes concerning the implementation of this training within the health faculty of the UPC, we chose not to do a purposive sampling but to systematically ask all the students that met the criteria. In practice, the 5 researchers-instructors mentioned the study to all the students at the end of every MHFA training they did, then the principal investigator (JS) contacted them through e-mails presenting in detail the study design and met with all the students willing to participate. Sample size was not defined in advance but was determined by data saturation according to the principles of “information power” (30) —based mostly on the criteria *the quality of dialog during the interview*, *the aim of the study* and the *sample specificity*. Inclusion of new participants continued until the analysis of new material no longer yielded new findings; that is, data collection and analysis were complete when the research group considered that the axes of experience obtained provided a sufficient explanatory framework for the data collected (31).

**Table 2:** Inclusion and exclusion criteria.

Inclusion criteria	Exclusion criteria
Age: 18 years or older (no upper limit) Student in health science in the university (medicine, pharmacy, nurses’ sciences, odontology) Have completed the 2-day MHFA training Able to communicate in French	Age: < 18 years Was partially absent during the 2-day training

### Stage 4: data collection, access to experience

One-to-one semi-structured interviews using an open-ended approach (32) were conducted online by the main investigator (JS), a child & adolescent psychiatrist expert in qualitative methods, without any prior established relationship with the participants. The interviews were structured by areas of exploration (Table 3) collectively determined by the group, from listening and reading two pilot interviews, not included in the final data. The interviews lasted 35–45 min. They were audio-recorded and transcribed into anonymized transcripts, including the participants’ expressive nuances.

**Table 3:** Interview guide.

Area of exploration	Potential questions
1. Before the training	What led you to take this training course? How did you learn about it? What were your views about mental health issues and psychiatry?
2. Lived experience of the 2-day training	How would you tell a friend about what happen during these two days? What is your most vivid memory?
3. Knowledge/literacy	What did you learn? What kind of knowledge, of skills did you gain? What was missing according to you?
4. Outcomes/effects	What did you gain from this training? You are now a MH first aider, what are you going to do or have you already done with this new status and skills?
5. Stigmatization/awareness	What about stigmatization about mental disorders? What is your position now?

### Stage 5: data analysis, from the description of the structure of experience to practical implications

The analytic procedure followed the IPSE approach (26). The IPSE analytic process is a rigorous procedure that relies on an inductive, phenomenological method. In practice, the analysis had two stages: one stage of independent work by two researchers, aided by Nvivo 12 software, and the other by the group, pooling the data collectively. In the individual procedure, the three qualitative researchers independently and simultaneously conducted a systematic descriptive analysis to convey each participant’s experience. This involved for each interview: (i) listening to the recorded interview twice and reading it three times; (ii) exploring the experience word by word, that is, cutting up the entire text into descriptive units; (iii) regrouping the descriptive units into categories. These stages are carried out with the help of QSR NVivo 12 software. During the group process, the three researchers met regularly with the other group members, who had familiarized themselves with the data by listening to and reading all the interviews as many times as necessary. These two-hour meetings began after the analysis of five interviews. The first set was intended to conduct the structuring phase, that is, to regroup the categories into axes of experience, constructed such that each could be linked to its subjacent categories, and then to determine the structure of lived experience characterized by the central axes. The second set of meetings covered the practical phase, the process of triangulation with the data in the literature that made it possible to identify the original aspects of the results and to suggest potential practical implications for the implementation of this training.

We used several criteria to ensure the rigor of the analysis and the trustworthiness of the results: triangulation, attention to negative cases and reflexivity within the group process. Reflexivity—the researchers’ reflection of their role in the study and its effects on their findings at every step of the research process—was worked on constantly in the group, during open discussions between the researchers.

## Results

The study included 20 students (16 women, 4 men). No students that agreed initially to participate to the study dropped out. Table 4 summarizes their characteristics. Their ages ranged from 19 to 26 years; 12 were in medical school, 5 in pharmacy and 2 in nursing sciences and 1 physiotherapy. The interview took place between 1 month and 9 months after the 2-day training.

**Table 4:** Participant characteristics.

	Age	Sex	Studies	Year of study	Personal psychiatric history	Family psychiatric history	Time since MHFA training (Months)
P1	25	F	Medicine	7th	burnout	Sister-depression	3
P2	21	F	Nursing sciences	2nd		Uncle-schizophrenia	1
P3	26	F	Medicine	3rd	Depression		1
P4	24	F	Physiotherapy	4th			2
P5	21	F	Medicine	3rd			3
P6	19	F	Medicine	2nd	Depression	Sister-mood disorder	4
P7	25	F	Pharmacy	5th	Bipolar disorder	Uncle and grandfather- alcohol use disorder Aunt- bipolar disorder	5
P8	19	F	Medicine	2nd	Depression	Brother-depression	1
P9	24	F	Pharmacy	5th		Mother-mood disorder	3
P10	24	F	Medicine	4th			2
P11	20	F	Medicine	3rd			3
P12	20	M	Medicine	3rd			1
P13	21	F	pharmacy	3rd			4
P14	22	M	Medicine	3rd		Uncle- non specified	3
P15	24	F	pharmacy	5th	Anxiety disorder		4
P16	21	M	Medicine	3rd			4
P17	20	F	Nursing sciences	2nd		Uncle, aunt- mood and psychotic disorders	3
P18	22	F	Medicine	4th	Depression and panic disorder		2
P19	22	F	Pharmacy	3rd	Panic disorder	Aunt-depression	5
P20	22	M	Medicine	5th			9

**Figure 1:**
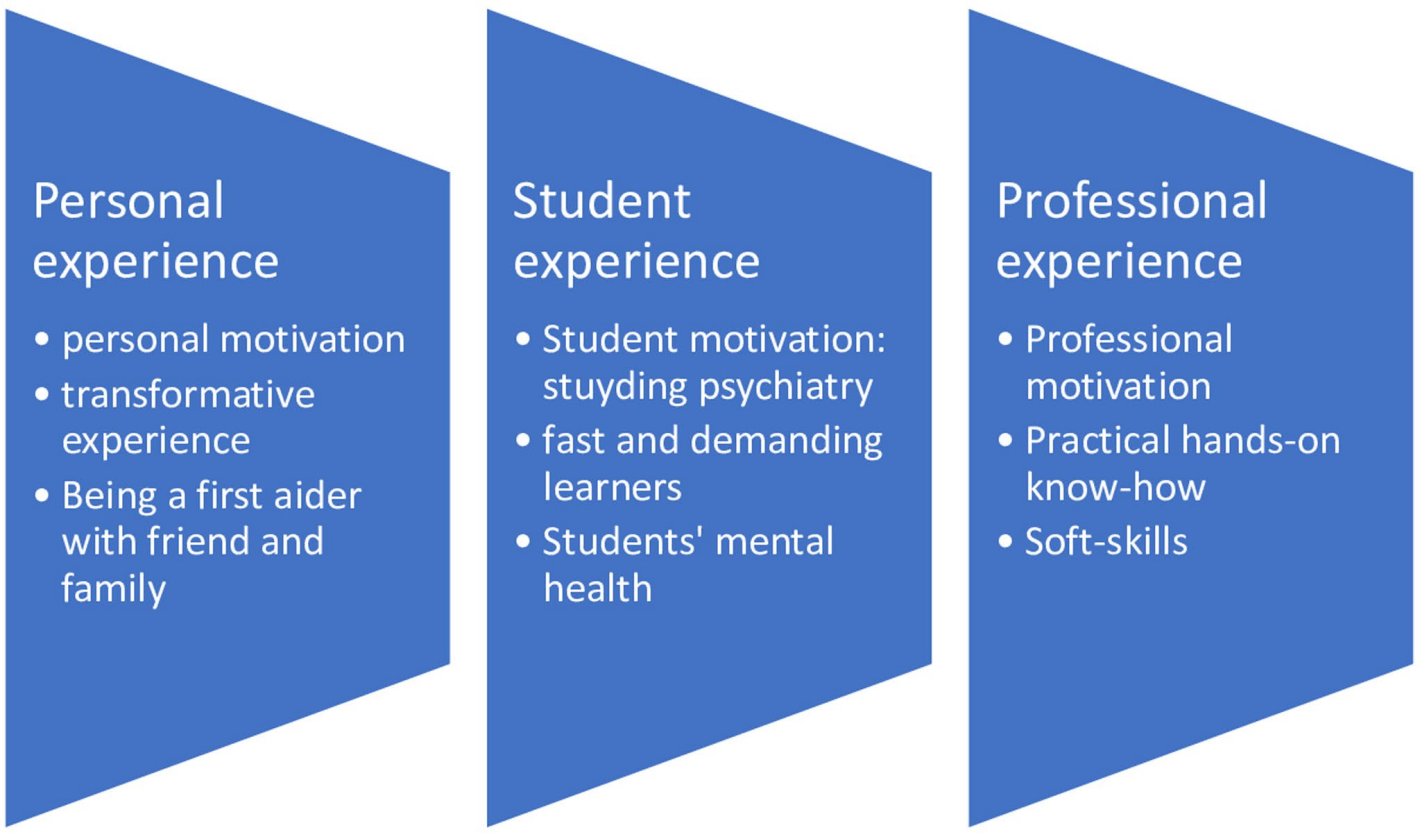
The structure of experience.

Data analysis produced a common structure of lived experience based on three central axes of experience: (1) a personal experience; (2) a student experience; and (3) a professional experience (Figure 1). Transcript excerpts presented below have been selected to exemplify the themes described and translated into English for the sole purpose of this article.

### A personal experience

#### A personal motivation

All participants said they were already aware of mental health related issues. Choosing to take this training course was directly linked to a personal involvement and motivation. More than half of the participants were directly concerned, either personally (*N* = 3), or through a family member (*N* = 3), or both (*N* = 5). Through this training, they were seeking to take better care of themselves and their loved ones.

*P3 "a personal motivation to help myself in difficult times."*

*P1 "I was facing this this with my little sister, she's hermetic, and so I needed to find ways of approaching her…*"

The other participants, without being directly concerned, explained that they had a long last interest about this thematic.

*P17 "Mental health training: I was immediately interested (…) just by the title, I knew I was going to like it, I wanted to know more."*

Finally, others saw it as part of their civic responsibility, a moral duty.

*P12 “It’s a feeling of duty done, I mean as a citizen, I’m doing my duty.”*

The importance of this personal motivation was so preponderant that one participant wondered about the course impact without such motivation.

*P15 "People who don't volunteer to take part, they won't be receptive, I don't know what they'd retain, I guess, it would still be better than nothing."*

#### A transformative experience

Most participants considered this two-day training course to be a meaningful experience that left lasting impact on them and their views on mental health in general, and on psychotic disorders and suicidal crises in particular. Participants described a “deconstruction” (P1) of preconceived misconceptions, specifically on these two topics.

*P3 “The way I’ve seen it before, psychiatry is for the crazy people, both the real ones and the fake ones, the one that need to invent problems for themselves.”*

*P5 “I think I had some prejudices that I wasn’t aware of.”*

Some participants recounted how MHFA training had helped them correct stigmatizing ideas they had about psychotic disorders, the disorders most misunderstood by students.

*P19 "I didn't know anything about psychotic disorders, I mean, I knew what everyone thinks they know about schizophrenia, which is bullshit! Well, now I know it's all bullshit, the stories about the dangerous, violent madman and all that."*

For some, however, this remained a boundary they could not cross.

*P9 "I don't feel too ready to intervene as a first aider… a psychotic crisis I don't feel at all ready to deal with, depression yes, anxiety attacks it’s fine too."*

Similarly, some participants reported the prejudices and stigmatizing ideas of other students about schizophrenia during the course.

*P1 "I was quite surprised to see that many had deep-rooted prejudices, all these false beliefs about schizophrenia.”*

*P7 There's one thing that struck me, there was this student who said " yes but schizophrenia is another level” and I said to myself it's an absolute failure of this training, schizophrenia It's the most stigmatized disorder, he's there so he feels concerned, but only for depression, anxiety, but when it comes to schizophrenia, that's when things get stuck.”*

They considered the suicidal crisis as the paradigmatic application of the MHFA, and described a shift in their attitude, several explaining “dare to ask the question now” (P1, P16, P18), or “ask again if there are suicidal ideas”(P8)-.

*P20 “when I learnt that you had to ask directly the question about suicidal thoughts, I wasn’t expecting it at all, I thought it was exactly the other way around, that you were supposed to turn around without saying it, my preconceived idea was completely shattered.”*

In general, many felts more empowered to act, and spoke about an increased capacity and willingness to engage with people with potential mental health issues.

*P4 "I don't know if I'm ready to be a mental health first aider, but I'd take the step of reaching out more easily."*

*P6 "I've understood that listening means keeping quiet, leaving moments for the other person to really talk and tell me things."*

*P20 the question “how are you”, I ask differently now, I really mean what I ask, I say “how are you feeling lately”. And I ask it even to myself.»*

#### Being a first aider with friends and family

Many participants did not have the opportunity to put their Mental Health First Aider status into practice. Yet, they were already “advocates”, correcting their loved ones when they made stigmatizing and discriminatory remarks.

*P2 "I told my uncle that it had nothing to do with laziness, that it was a symptom of his depression."*

Others had already intervened with a friend or family member with a chronic disorder, with the aim of encouraging them to seek help from professionals.

*P20 "My friend thanked me, saying that without me he wouldn't have gone to see a shrink, that's a personal satisfaction!"*

If this objective was not achieved, they noted a change in their interactions with this relative.

*P1 "I tried it with my sister, it wasn't too conclusive, but still by listening to her and comforting her, her attitude changed, it worked better."*

Finally, one participant recounted assisting a friend in suicidal crisis at a party, experiencing the practical value of what she had learned in the training.

*P11 "If I hadn't done the training, I wouldn't have succeeded, but there I was the only one who really went all the way and asked the questions about suicide! And having talked about it with friends, they said to me 'but I don't know how you managed to ask the right questions (…) from the moment I said it, it switched…; it broke a taboo and he really talked."*

### A student experience

#### Student motivation: studying psychiatry

Many of the participants, particularly the medical students, decided to take this course to learn – for younger students – or revise – for more advanced students – psychiatry, that is the academic course in their curriculum.

*P16 "I'm in my third year, we only had a few neuropsychiatry classes last year, but I'd revised more the neurology part, which was more difficult, so I really didn't know much, and here in two days, I got ahead of the program."*

*P12 “It's for the general population, so I knew it wouldn't be detailed, it wasn't a psychiatric training course, but it's always good to have a reminder.”*

Taking this MHFA training course was motivated by a lack of courses on mental health and psychiatry in their academic curriculum.

*P5 "Honestly, we hardly have any courses on mental health or psychiatry in the first three years, so as soon as I see an option or training on the subject, I go for it."*

Students then expressed frustration with the theoretical content of the MHFA course; they would have liked to know more, to have gone into greater depth on aspects not covered by the training.

*P8 "I was expecting something more medical, a bit more medical gestures, a bit more explanations, more in-depth explanations of mental disorders."*

*P13 “It was good for a first approach, but I would have liked to have seen a little more, a little more in-depth (…)to know more about the underlying mechanisms of the disorders.”*

Similarly, they would have liked a pedagogical summary or an exam to assess their knowledge.

*P17 "I would have liked it if, at the end, we could have gone over the key points together, the essential notions, that would have helped me integrate them."*

*P19 "I was expecting a quiz or an exam, something to confirm or validate what we'd learned."*

#### Fast and demanding learners

Students emphasized both their ability and habit to learn and assimilate content quickly, hence a certain frustration not to have learned more.

*P8 “In medicine, we are used to learning fast, learning well and learning a lot.”*

*P10 “We medical students are used to a higher flow rate, so there was a certain frustration not being able to go further.”*

Repetition of the same messages and memorization exercises, particularly the acronym listing the steps and actions in the MHFA plan, were seen as useless and a waste of time, even infantilizing for a few, undermining the pedagogical power of the course.

*P18 "We had to repeat, mime and draw “AERER”(the French acronym of the MHFA plan)… even though we had all understood it straight away… and then we didn't have time to answer more specific questions."*

Most students valued the course’s active and interactive pedagogy.

*P20 “It wasn't a top-down course, you had to stand up and move a lot, exchanged ideas, took part, got involved; that was so stimulating (…)”*

#### Students’ mental health

Very few students spontaneously addressed the issue of students’ mental health. When the interviewer did, many said that the course content was not sufficiently adapted to the specific suffering of students. Even if anxiety, depression, and suicide were well addressed and seen as the main issues they had observed in the student environment, others of equal importance to them were missing, namely eating disorders, self-mutilation and sexual and gender-based violence.

*P1 "I would have liked to see a focus on eating disorders, as they are very common among students.”*

Although the issue of sexual and gender-based violence was beyond the scope of this training course, several students felt it was essential to address this issue within the two day-course anyway.

*P10 "The only issue not tackled is sexual and gender-based violence, but it's not completely in the scope (…) the section on aggression focuses on physical aggression, whereas students experience more moral or sexual aggression… Being a woman and talking to my female student friends, I can tell you this issue is essential and should be in the course."*

Finally, participants raised the question of the student group that had formed over the 2 days, with a wide range of experiences, from a lack of ties and interactions to a strong sense of belonging and the prospect of mutual aid within the group.

*P5 "We weren't much of a group. At lunchtime, people scattered, people didn't eat together, at times when there was no training, we were free electrons."*

*P10 "We were very interactive, we were a group, and we were able to really talk; in fact, we've kept in touch and we've already said we could help each other if needed."*

### A professional experience

#### A professional motivation

In addition to the personal and student motivations already mentioned, several participants reported a significant event during their internships at the hospital that led to their decision to take this training course. For one, it was an unsettling encounter with a patient with psychiatric disorders with difficult interactions both with the patient and the medical staff. For others it was a supervising health professional who recommended the training.

*P11 "During my nursing internship in orthopedic surgery, I went to talk to a patient who had psychiatric issues, but I hadn't been told. He started reacting strangely, and I was taken by surprise, so I left the room and cried because I didn't know what to do (…) "plus the doctors blamed me a bit, saying, your studies are going to be tough, so why are you crying?*

*P10 "During my internship, a patient killed himself and the doctor told me it might be interesting to do this training."*

Most of the participants aimed, through this course, to be able to interact better with their patients, as part of their internship or in their future professional practice.

*P2 “If I was faced with a patient in crisis, I felt I did not have the tools.”*

Once again, this professional motivation was accompanied by frustration, as the training was not tailored for healthcare professionals. As future healthcare professionals, the students found it absurd that they were taught how to “encourage appropriate professional help,” but not what professionals do after.

*P3 "It's biased because I'm a medical student, so I admit I was a little frustrated, I was left wanting more… We're told how to direct them towards professionals, yes, but the professionals are us, well tomorrow, but I did understand that it was for any audience, so that's normal."*

*P6 "We were learning to be first-aiders and not doctors, but it was often frustrating."*

#### Practical hands-on know-how

The fear of doing things badly or not knowing how to do things was a recurring theme.

*P5 "I was a bit afraid, afraid of making mistakes and triggering reactions… and afraid of not being able to help, of not having the tools (…) either to do and do badly, or not to do, which is no better."*

All the participants then emphasized the practical, concrete knowledge they had acquired over the two-days course, and the know-how they had acquired. They specifically mentioned “communication tools,” “relational techniques,” and “phrases to say and those not to say.”

*P10 "learning concrete things is essential for our future work, words not to say, words or questions not to avoid… that's really what I was looking for in this training."*

*P3 "This training showed me 'how to do it'… how to approach these people…"*

At the end of the training, many felt ready to apply this know-how in internships with patients more than in their everyday lives.

*P10 "In the street, not so much, but in my internships at the hospital, I feel ready, I feel ready to talk to a patient about these subjects."*

Similarly, they valued the concrete, “real-life situation” aspect of their training, which compensated for a gap in their academic teaching, making a distinction between learning about the disease as a theoretical object but not about person that is sick.

*P5 "We have psychiatry courses, and it's good to have all the theory, but we don't have an illness in front of us, we have a patient, a human being."*

Several participants also recognized that this concrete interactional know-how was more difficult to transmit and teach than the theoretical courses to which they were more accustomed to.

*P9 "practice is more complicated, it's more difficult than acquiring theoretical knowledge, plus we are so used to retaining theoretical knowledge in our courses."*

*P4 "in the end, this course is mainly designed to teach us communication and listening skills, so it's harder to pass on and put into practice."*

For some students, the transmission of this know-how would have required more practical training, for example by doing more role-playing so that everyone could experience during the course what it is to act as a mental health first-aider. But others felt that the training was balanced in its theory/practice ratio, and that adding more practice would be at the expense of the reflection and theoretical knowledge needed by the mental health first aider.

*P10 "One role-play per person so that everyone has experienced it, for me that would be the basis."*

*P11 "It's not as practical as I'd imagined, but on reflection, it couldn't be much more practical."*

In fact, this was the major difference raised by participants between somatic first aid and MHFA, which for them required more thought.

*P20 “(Somatic) First aid is about reflex, cardiac arrest equals massage, you don't ask yourself questions, MHFA isn't about reflex, it's about reflection,…the plan, the acronym you can't apply it stupidly, you have to think, and for that you need to know enough about mental disorders.”*

#### Soft skills

Many students felt that this training had also had an impact on their interpersonal and soft skills. First and foremost, it was a question of ethical posture in the relationship with the people they would be helping. They emphasized notions such as availability and respect for others.

*P3 “Being available, saying ‘I’m here’, that’s what I learned from this training.”*

Participants who were already in contact with patients talked about how they had integrated this “savoir-être” (know-how-to-be) with their patients.

*P1 "I feel more at ease addressing these issues, and I do so with my patients (…) I've understood that listening means keeping quiet, leaving moments for the other person to really talk and tell me things."*

The others projected that these soft skills would shape their future professional identity.

*P10 "I think it's important for future healthcare professionals to have already integrated a posture, a way of acting and interacting with patients, a way of doing things."*

## Discussion

Some findings of this study are in line with some MHFA training outcomes reported in the literature, such as the improvement of students’ awareness, knowledge, and literacy about mental health, as well as the development of empowerment to act (13–16, 19, 23, 24). Yet, those aspects were secondary in the participants narratives and the three axes – personal, student and professional – structuring the experience of MHFA training of these healthcare students go beyond the sole improvement of skills, knowledge, and awareness, and draw original insights for this specific population.

Regarding the students’ motivation, our findings show a dual intrinsic motivation with strong self-determination (33), one associated with the past – the personal motivation due to familiarity or a long-standing interest on the matter – the other related with the future – the professional motivation linked to the construction of professionalism among these future healthcare professionals-. Since this training was optional, it is quite logical to find only intrinsic motivation in these students. This is a limitation of our study as we cannot transpose our results to students for whom this course would have been mandatory. Yet, motivation is not just about why a student engaged in taking this 2-day course, but also how he/she got involved during the course and persevered afterwards. Last year, two medical students wrote an editorial in *Academic Medicine* to advocate the urgent inclusion of MHFA training in medical curricula, with the same parity as the physical first aid training (34). Making the course part of the curriculum would add some extrinsic motivation but we think that, even as a mandatory course, it would still be essential to address the students’ motivation to become mental health first aiders.

In our results, there was not motivation based on the *here and now,* that is a motivation from the present student context, apart from a misuse of the course to learn and revise psychiatry. Our results suggest that the content of the course should be more tailored for the student population and that a specific MHFA training developed for this population will increase students’ motivation and engagement. This would be a way to contextualize and situate learning (35), which could not only increase students’ involvement but also the appropriation of course content by linking it to a relevant context, the student context. This is consistent with the development of other MHFA courses that are variations of the standard MHFA course, targeting specific populations such as professionals working with youth or adolescents (36). Based on our findings, such specific MHFA course should include content about frequent symptoms and disorders – self-injuries, eating disorders- but also specific factors such as sexual and gender-based violence. Indeed, a recent study (5) conducted in UPC, humiliation, sexual abuse and sexual harassment were identified as associated factors of major depressive episode.

A course with content closer to what students are experiencing in terms of mental health would better ensure that students acquire comprehensive skills for early intervention with their peers, that remains the main objective of MHFA training within this population. Our results suggest that the misuse of this course to learn or revise psychiatry could be another obstacle to this main objective. This points to the need for earlier and more teaching on mental health, psychiatry, and health psychology in the health studies curriculum. In order to avoid this misuse and confusion, the student MHFA training course should be included in a global and multimodal program led by each university rather than a stand-alone initiative, so to better address students’ mental health needs such as policies to strengthen the mental health of students in courses; developing mental health care programs or consultations within the university; local communication strategies to break the silence and the stigma about mental health disorders among students and promoting well-being; and monitoring continuously the mental health needs of students (37–39).

Furthermore, educational format may be refined regarding specificity of healthcare studies. MHFA training has been conceived to address general population, ensuring a basic level for every people interested in mental health aid., hence a feeling of frustration among those students willing to know more and aid further. Similarly, students in our sample did not consider starting mental first aid interventions with their peer students, but both within their personal relationships and at work as future healthcare professionals. They are in fact future healthcare professionals, and this is what motivate them to acquire the practical knowledge and soft skills so they can address mental health issues with their patients. Here again, it may appear as a misuse, as this training is not tailored for healthcare professionals. However, beyond the need to integrate into their curriculum courses focusing on professionalism, soft-skills, and patient-caregiver communication, we believe it would also be necessary to contextualize this MHFA course for healthcare students by considering the professional field, internships at the hospital, as a privileged space for the transfer of learning. Transfer of learning is the use of knowledge, skills and abilities learned in training into a work situation (40). Indeed, our results suggest that students would be more inclined to apply the skills learned in this course within their internship, thus benefiting from the help and feedbacks from supervising professionals. It would also clarify the paradox of MHFA for this population, that is to be limited to “encourage appropriate professional help” while being soon-to-be healthcare professionals themselves.

Different levels of interventions should be elaborated to start from a MHFA attitude during the first clerkships, to then developing progressively a healthcare professional attitude, while keeping the core principles of MHFA.

## Limitations

This qualitative study has some limitations that must be considered. First, qualitative findings are contextualized, and this study took place in France, in a Parisian university; caution is therefore required in transposing our findings to other places. Second, as mentioned above, participants were students who chose voluntarily to take this course and among them, 40%, self-declared a personal psychiatric history, a slightly higher rate than the one found in this specific population (3, 4). Our findings are therefore contextualized within a specific concerned population of students. This is not a bias in qualitative research but this is a limitation, impeding the transferability of results to other students unaware of and unconcerned by the subject of mental health.

Plus, as already mentioned, in order to rapidly refine the course, we decided not to use a purposive sampling strategy and only recruit students that show an interest to be interviewed. As a result, our sample is in fact more representative than exemplary of the population of these volunteered students who undertook the course. Indeed, our sample is not balanced between the different types of healthcare students – 60% of our participants being medical students and 25% pharmacy students- with potentially more weight for these students in the results. However, on that matter, our sample is representative of the 153 students trained at the time of recruitment: indeed, 83 were medical students (54%), 39 were pharmacy students (23.5%)- only 16 were in nursing sciences (10%), 4 in physiotherapy, 6 in midwifery and 5 in odontology. Further studies should focus on each healthcare profession to explore the specific aspects non-covered by our results.

Yet, we consider that we were able to reach data saturation among this specific, concerned, population. Third, subgroup analysis of male students and women students did not reveal any differences between them in relation to the results, although gender differences have been described in terms of prevalence of mental disorders and mental health risks (41), and professional adversities in the field (42). Finally, without having prior contacts with the students interviewed, the research team belongs to the same university and the three psychiatrists are also faculty members, it might have impacted the data collection.

## Conclusion

These results of this study directly question the need to develop a specific MHFA training course directly addressing students’ mental health issues and related issues, such as gender-based and sexual violence as well as educational format better aligned with students’ level and healthcare training modalities. When delivered to future healthcare professionals, this course should be integrated into the curriculum and the internships so students can apply and use MHFA skills not only as citizen with their friends, family, and peers but also as healthcare professionals with patients. This would enable a better transfer of learning and at the same time participate to reduce stigma of mental illness among healthcare professionals. In practice, based on our findings, we would recommend to, first increase the amount of mental health and psychiatric courses given during the first years of healthcare studies; then to integrate into the curriculum, during the first or the second year, as an optional course this 2-day MHFA course with an adaptation of the content closer to what students are experiencing in order to improve self-protection and help among peers; and finally to create a level 2/advanced MHFA course specifically tailored for present and future healthcare professionals.

## Data availability statement

The raw data supporting the conclusions of this article will be made available by the authors, upon reasonable request.

## Ethics statement

Ethical approval was not required for the studies involving humans because the research complies with French regulations governing observational research involving students (declaration of compliance with the CNIL reference methodology MR004 and entry in the register of such research hosted by Health Data Hub website). The study was conducted in accordance with the local legislation and institutional requirements. The participants provided their written informed consent to participate in this study.

## Author contributions

JS: Conceptualization, Formal analysis, Investigation, Methodology, Writing – original draft. PE: Formal analysis, Validation, Writing – review & editing. TB: Formal analysis, Funding acquisition, Validation, Writing – review & editing. YD: Investigation, Validation, Writing – review & editing. M-AP: Formal analysis, Methodology, Supervision, Validation, Writing – review & editing.
